# The use of income information of census enumeration area as a proxy for the household income in a household survey

**DOI:** 10.1186/1478-7954-7-14

**Published:** 2009-09-22

**Authors:** Fabio S Gomes, Mauricio TL Vasconcellos, Luiz A Anjos

**Affiliations:** 1 Escola Nacional de Ciências Estatísticas, Fundação Instituto Brasileiro de Geografia e Estatística, Rua André Cavalcanti 106, Sala 403, Bairro de Fátima, 20231-050 - Rio de Janeiro, RJ, Brazil; 2 Área de Alimentação, Nutrição e Câncer, Coordenação de Prevenção e Vigilância, Instituto Nacional de Câncer, Rua dos Inválidos 212, 4° andar, Centro, 20231-048 - Rio de Janeiro, RJ, Brazil; 3 Laboratório de Avaliação Nutricional e Funcional, Departamento de Nutrição Social, Universidade Federal Fluminense, Rua Mario Santos Braga 30, Sala 415, Campus do Valonguinho, 24020-140 - Niterói, RJ, Brazil; 4 Escola Nacional de Saúde Pública Sergio Arouca, Fundação Oswaldo Cruz, Rua Leopoldo Bulhões 1480, 21041-210 - Rio de Janeiro, RJ, Brazil

## Abstract

**Background:**

Some of the Census Enumeration Areas' (CEA) information may help planning the sample of population studies but it can also be used for some analyses that require information that is more difficult to obtain at the individual or household level, such as income. This paper verifies if the income information of CEA can be used as a proxy for household income in a household survey.

**Methods:**

A population-based survey conducted from January to December 2003 obtained data from a probabilistic sample of 1,734 households of Niterói, Rio de Janeiro, Brazil. Uniform semi-association models were adjusted in order to obtain information about the agreement/disagreement structure of data. The distribution of nutritional status categories of the population of Niterói according to income quintiles was performed using both CEA- and household-level income measures and then compared using Wald statistics for homogeneity. Body mass index was calculated using body mass and stature data measured in the households and then used to define nutritional status categories according to the World Health Organization. All estimates and statistics were calculated accounting for the structural information of the sample design and a significance level lower than 5% was adopted.

**Results:**

The classification of households in the quintiles of household income was associated with the classification of these households in the quintiles of CEA income. The distribution of the nutritional status categories in all income quintiles did not differ significantly according to the source of income information (household or CEA) used in the definition of quintiles.

**Conclusion:**

The structure of agreement/disagreement between quintiles of the household's monthly per capita income and quintiles of the head-of-household's mean nominal monthly income of the CEA, as well as the results produced by these measures when they were associated with the nutritional status of the population, showed that the CEA's income information can be used when income information at the individual or household levels is not available.

## Background

The place of health on the international agenda for development has been broadened [1] and health inequalities between and within countries have become a topic of great interest [2-5]. The concept of health inequalities includes the presence of unfair, avoidable, or remediable health differences among populations or specific groups defined according to social, economic, demographic or geographic criteria [6]. It implies a failure in avoiding or overcoming these differences that overlooks basic human rights [7].

For these reasons, it is common that population surveys collect socioeconomic information when the purpose is either exploratory or descriptive (and this information becomes the main focus) and socioeconomic information is associated with outcomes or other variables of interest.

Income and education are the most used variables to characterize and/or discriminate among socioeconomic groups. However, the collection of this information, particularly income, is sometimes difficult and can be influenced by other factors in population-based studies. These interferences may result in either total failure to obtain it or misreporting (under- or overestimation) [8].

In Brazil, the Census Enumeration Areas (CEA) are used to assess the data of the Brazilian Demographic Census but they are also used as conglomerates of households for other population-based surveys. They are defined as contiguous groups of approximately 300 households respecting administrative and political boundaries and identified by stable and easy location reference points [9]. Some of the CEA's information may help in planning the sample of such studies but it can also be used for some analyses that require information that is difficult to obtain at the individual or household level, such as income.

Although the use of this kind of information would be especially useful in developing countries, the few available studies in this area found in the literature were conducted exclusively in high-income countries in North America or Europe and in Australia [10-19]. There are remarkable differences in the methods of these studies, such as the definition of area levels, the independent and outcome variables adopted, and the statistical analysis, all of which hinder detailed comparisons. Most studies are interested in substituting individual-level [10,12,13,15-17,19], and more rarely household-level [14], information by the area level available in the census. Variables used to describe socioeconomic status vary from self-reported income data to socioeconomic scales. There is a variety of health outcomes in the analyses that make it difficult to generalize [10]. Furthermore, the studies analyze the data using different procedures such as factor analysis, log-linear models, and estimation of correlation, agreement and reliability indexes such as intra-class correlation, Cohen's kappa or Kendall's coefficient of concordance. As a result, it is difficult to explain or predict the situations for which the different levels of socioeconomic information (e.g. CEA, household, individual) would produce similar results.

Additionally, no study has empirically compared the trade-offs in terms of cost savings, potential bias or loss of accuracy due to the use of area-level instead of individual- or household-level information. It has been suggested that the census-aggregated information is complementary because it may have a different construct meaning, depending on how it is defined in association with the health outcomes [10]. In middle- and low-income countries, savings would probably surpass bias and accuracy costs.

The gap between the year of the census (every 10 years in Brazil) and the year of a given survey may play a crucial role in the socioeconomic characteristics of the population. Additionally, the fact that some countries' economic growth may be stationary or there is very discrete social mobility may facilitate the comparisons because there may not be expressive changes in family or individuals' income or socioeconomic status between the year of the census and the survey. On the other hand, if the country's economic growth is reflected in individual and family income, one may not be able to use the census information.

The purpose of the present study was to assess the validity of household income data from CEA to represent household income obtained in a household survey. In practical terms, it sought to verify if the CEA income information could be used as a proxy for household income in a household survey conducted to assess the nutritional status of the population of Niterói, a city in the state of Rio de Janeiro, Brazil.

## Methods

The Nutrition, Physical Activity and Health Survey (*Pesquisa de Nutrição, Atividade Física e Saúde *- PNAFS) was the first household survey conducted to assess the nutritional status and health conditions of adolescents and adults living in Niterói, Rio de Janeiro, Brazil. Data collection was carried out between January and December 2003. Niterói is located in the metropolitan region of Rio de Janeiro that had 459,451 inhabitants in 2000, according to the last Brazilian census [9].

To guarantee the representativeness of the population of Niterói, a probabilistic sample of households was designed. The households were selected from the 2000 population CEA listing (there are 696 CEA with an average of 216 households per CEA in Niterói) [9] in two stages (CEA and household).

In the first stage, 110 CEA were selected, systematically, with probabilities proportional to the number of permanent private households. Prior to selection, the CEA were ordered from lowest to highest according to the head-of-household's mean nominal monthly income, thus implicitly stratifying the CEA by mean income and ensuring the selection of CEA from all income levels.

In the second stage, 16 households were selected in each CEA with equal probability, using an inverse sampling procedure [20], analogous to that applied in the World Health Survey in Brazil [21], leading to a sample size of 1,734 households (3,619 subjects) after the refusal of 26 households to participate in the study.

The sample weights were calculated as the product of inverse selection probabilities in each stage, using the estimator proposed by Haldane [20] adapted to be used in household surveys [21]. To reduce selection biases, common in household surveys, the natural sampling weights were calibrated to provide estimates that coincide with known population totals [22].

The calibration post-strata were defined using the variables age and sex. The combination of the two categories of sex (male and female) and age -- categorized as seven brackets: 10-19.9; 20-29.9; 30-39.9; 40-49.9; 50-59.9; 60-69.9; and 70 years or more -- resulted in 14 post-strata (2 sexes × 7 age brackets). For the calibration of sampling weights, the household natural weight (*W*_*ij*_) was multiplied by a calibration factor (*g*_*ij*_), providing the household calibrated weight , where *i *represents the index of the selected CEA, *j *the index of the selected household and *d *the 14 post-strata domains, as indicated above. The Generalized Regression Estimator proposed by Deville & Särndal [23] was adopted to estimate the calibration factor *g*_*ij *_as , where *q*_*ij *_is a constant usually defined as 1 [22], **x**_*ij *_represents the vector of auxiliary variables (i.e., sex and age), **t**_*x *_denotes the vector of known population totals, and  the vector with the estimates of the auxiliary variables calculated using natural sample weights.

Despite its extensive use, the Cohen's Kappa index (***κ***) does not provide information about the agreement/disagreement structure of data and it cannot be used to analyze ordinal scale categories [24], such as education or income strata. For this reason, the adjustment of uniform semi-association models was performed in the present analysis [25]. It is a generalized linear model of the *Poisson *family with *log *link function that considers the ordination structure of the variable's categories. Three components of the structure of agreement and disagreement compose this model: (1) the agreement at random; (2) the agreement due to the association between classifications; and (3) the agreement after eliminating the effects of the agreement at random and the association between variables [25,26]. Beyond the combination of the effects of agreement and disagreement, the semi-association model also considers the variations by categories, in the main diagonal, different from other models that assume the agreement is the same for each cell in the main diagonal [25,26].

Besides the adjustment of the model that assesses the agreement/disagreement between the classification of income categories defined according to the CEA or the field-obtained information on household income, two other models were adjusted: (1) the information on household income from male-headed and (2) female-headed households. This was motivated by the hypothesis that when the woman is the head of the household, she may not know exactly her spouse's income and vice versa. Therefore, household income might be estimated with different errors if the head of the household knows or does not know the spouse's income.

The adjusted model agreement grades were estimated for each cell in terms of *odds ratio *(OR), using the measure *τ*_*ij *_(where *i *indicates the line and *j *the column of the cell) as proposed by Darroch & McCloud [27].

In addition to the adjustment of the model, Cohen's weighted Kappa [28], Kendall's coefficient of concordance [29], Krippendorff's alpha reliability coefficient [30] and Spearman's correlation *ρ *[31] were also estimated in order to check the robustness of the model and to allow comparisons to other studies.

To illustrate the comparison between the two income information applied to an epidemiologic study, the distribution of nutritional status categories of the population of Niterói (≥ 10 years of age) according to income quintiles (CEA and household) was performed.

To test the hypothesis that the distribution of the population by nutritional status categories according to the household income quintiles is equal to the distribution according to the quintiles constructed with the income of CEA the Wald statistic for homogeneity based in the sampling design was used [32].

Body mass and stature data were collected in the households and used to calculate the body mass index (BMI = body mass in kilograms divided by stature in squared meters) as described elsewhere [33]. BMI for age and sex was used to define the nutritional status of adolescents (10-20 years of age) using the cut-off points presently recommended by the World Health Organization (WHO): low-BMI-for-age/thinness (< -2 Standard Deviations), overweight (≥ 1 Standard Deviation) and obesity (≥ 2 Standard Deviations) [34]. For adults (≥ 20 years of age), the BMI cut-off points of < 18.5 kg/m^2^, ≥ 25 kg/m^2 ^and ≥ 30 kg/m^2 ^were used to define the categories of low-BMI/underweight, overweight and obesity, respectively [35].

The Institutional Review Board of the Sergio Arouca National School of Public Health of the Oswaldo Cruz Foundation approved all research procedures.

All estimates and statistics were calculated using the calibrated weights based on the structural information of the sample design, and a significance level lower than 5% was adopted. The analyses were conducted in R language and environment, version 2.6.1 [36].

## Results and Discussion

The parameters  represent the effect of a given quintile defined according to the household- or CEA-level income information on the *log *of the expected frequency of being categorized in the first quintile by both household- and CEA-level income information (), which was set as reference to conduct the comparison. As the quintiles defined according to household- or CEA-level information get more distant from the reference quintile (first), the expected frequencies of being categorized in the first quintile according to one of the two levels of information and at the same time of being categorized in the second, then in the third, then in the fourth and then in the fifth quintile according to the other level of information, significantly decreases consecutively. These parameters account for the share of the agreement that is due to random (Tables 1 and 2).

**Table 1:** Uniform semi-association model

**Parameter estimator**	**Parameter estimate**	**SE**	**p-value**
(intercept)	9.23	0.010	< 0.001
(2^nd ^quintile of household income^†^)	-0.45	0.013	< 0.001
(3^rd ^quintile of household income^†^)	-0.80	0.013	< 0.001
(4^th ^quintile of household income^†^)	-1.51	0.016	< 0.001
(5^th ^quintile of household income^†^)	-2.24	0.021	< 0.001
(2^nd ^quintile of CEA's income)	-0.57	0.012	< 0.001
(3^rd ^quintile of CEA's income)	-1.22	0.013	< 0.001
(4^th ^quintile of CEA's income)	-1.91	0.018	< 0.001
(5^th ^quintile of CEA's income)	-3.22	0.023	< 0.001
	0.31	0.002	< 0.001
	0.38	0.026	< 0.001
	0.51	0.024	< 0.001
	0.10	0.024	< 0.001
	0.81	0.022	< 0.001
	0.39	0.017	< 0.001

^†^Quintiles of household income defined as the household's monthly per capita income.

SE = Standard Error

CEA = Census Enumeration Area (quintiles of CEA's income defined as the head-of-household's mean nominal monthly income of the CEA).

**Table 2:** Uniform semi-association models adjusted using only household income information of households in which the head-of-household was male or female

	**Sex of the head-of-household**					
	
	**Male**			**Female**		
**Parameter estimator**	**Parameter estimate**	**SE**	**p-value**	**Parameter estimate**	**SE**	**p-value**

(intercept)	8.45	0.015	< 0.001	8.62	0.013	< 0.001
(2^nd ^quintile of household income^†^)	-0.33	0.018	< 0.001	-0.57	0.018	< 0.001
(3^rd ^quintile of household income^†^)	-0.73	0.018	< 0.001	-0.87	0.020	< 0.001
(4^th ^quintile of household income^†^)	-1.44	0.022	< 0.001	-1.62	0.025	< 0.001
(5^th ^quintile of household income^†^)	-2.11	0.028	< 0.001	-2.54	0.033	< 0.001
(2^nd ^quintile of CEA's income)	-0.70	0.015	< 0.001	-0.42	0.018	< 0.001
(3^rd ^quintile of CEA's income)	-1.48	0.018	< 0.001	-0.94	0.020	< 0.001
(4^th ^quintile of CEA's income)	-2.38	0.025	< 0.001	-1.45	0.025	< 0.001
(5^th ^quintile of CEA's income)	-3.90	0.033	< 0.001	-2.56	0.033	< 0.001
	0.36	0.003	< 0.001	0.27	0.003	< 0.001
	0.40	0.036	< 0.001	0.34	0.037	< 0.001
	0.94	0.032	< 0.001	-0.11	0.037	< 0.01
	0.09	0.034	< 0.05	0.10	0.034	< 0.01
	0.91	0.030	< 0.001	0.78	0.033	< 0.001
	0.60	0.024	< 0.001	0.14	0.025	< 0.001

^†^Quintiles of household income defined as the household's monthly per capita income.

SE = Standard Error

CEA = Census Enumeration Area (quintiles of CEA's income defined as the head-of-household's mean nominal monthly income of the CEA).

The parameters *β *and *δ*_*i*_(*i *= *j*, *i *= 1 ..., *i*), estimated by  and  measure the association and agreement, respectively, between the measures of ordinal classification of income. The estimates of these parameters are statistically different from zero (p < 0.001) (Table 1), which indicates that the assessments of income quintiles made by means of CEA and household information are not different and that the classification of households in the quintiles of household income tend to be associated with the classification of these households in the quintiles of CEA income. The agreement between measures ranges from 0 (no agreement) to 1 (perfect agreement). All agreement estimates () are significantly different from zero, which means that there is enough statistical evidence to reject the hypothesis that there is no agreement between the pairs of quintiles defined by household- and CEA-level information (Tables 1 and 2).

Table 2 shows the estimates of the cited parameters for male-headed and female-headed households. The uniform semi-association models adjusted for the distinct sexes has resulted in the same conclusion suggesting that the sex of the head of the household does not influence the structure of agreement/disagreement of the information of CEA and household income.

Based on the model presented in Table 1, the agreement grades  were estimated for each *ij *cell and its respective 95% confidence interval (95% CI) (Table 3). Adopting the first cell of the diagonal (*i *= 1, *j *= 1) as an example of interpretation, the value in the matrix's diagonal represents the *OR *of a household to be categorized in the 1^st ^quintile of income by the CEA income is two times greater ( = 2.1; 95% CI = 1.9-2.4) when the household is located in the 1^st ^quintile of household income. To illustrate the interpretation of values that are out of the diagonal, the cell located in line 1, column 5 was taken. In this case, the *OR *of a household to be categorized in the 1^st ^quintile of household income rather than in the 5^th ^quintile of household income is approximately 308 times greater ( =  = 307.9; 95% CI = 263.6-359.7), considering that the household, according to the CEA income, belongs to the 1^st ^quintile rather than the 5^th ^quintile.

**Table 3:** Symmetric matrix with the agreement grades estimates () for each cell, with its respective 95% confidence intervals (95% CI).^§‡^

	***CEA***				
	
**Income quintiles**	**1^st^**	**2^nd^**	**3^rd^**	**4^th^**	**5^th^**
*Household*^†^					
1^st^	2.1 (1.9-2.4)	3.3 (3.0-3.7)	5.6 (5.0-6.3)	53.7 (47.0-61.3)	307.9 (263.6-359.7)
2^nd^		2.8 (2. 5-3.0)	2.5 (2.3-2.8)	13.0 (11.6-14.4)	40.0 (35.4-45.1)
3^rd^			1.2 (1.1-1.4)	3.4 (3.1-3.7)	5.7 (5.1-6.2)
4^th^				5.1 (4.7-5.5)	4.5 (4.2-4.9)
5^th^					2.2 (2.0-2.3)

All  are significantly different from 1.

^§^***In the diagonal***,  represents the Odds Ratio of a household to be categorized in the same quintile by both household and CEA income.

^**‡ **^***Out of the diagonal***,  represents the Odds Ratio of a household to be categorized in the *i*^*th *^quintile of household income rather than in a different (*j*^*th*^) quintile of household income, considering that the household, according to the CEA income, belongs to the *i*^*th *^quintile rather than the *j*^*th *^quintile.

^†^Quintiles of household income defined as the household's monthly per capita income.

CEA = Census Enumeration Area (quintiles of CEA's income defined as the head-of-household's mean nominal monthly income of the CEA).

The values of  indicates the *OR *of assessment measures to be concordant rather than discordant. Observing the values in the first line (fixing line 1 and varying columns) or in the fifth column (fixing column 5 and varying lines) of Table 3, it is possible to note that the agreement increases as the quintiles get more distant from the others, according to the interpretation above (Table 3).

According to the agreement classifications more widely used [37,38], the estimated indexes indicate fair to moderate levels of agreement and are statistically significant: Kappa_*w *_= 0.49 (p < 0.001); Kendall's coefficient of concordance = 0.41 (p < 0.001); Krippendorff's alpha = 0.48; Spearman's *ρ *= 0.49 (p < 0.001).

**Figure 1:**
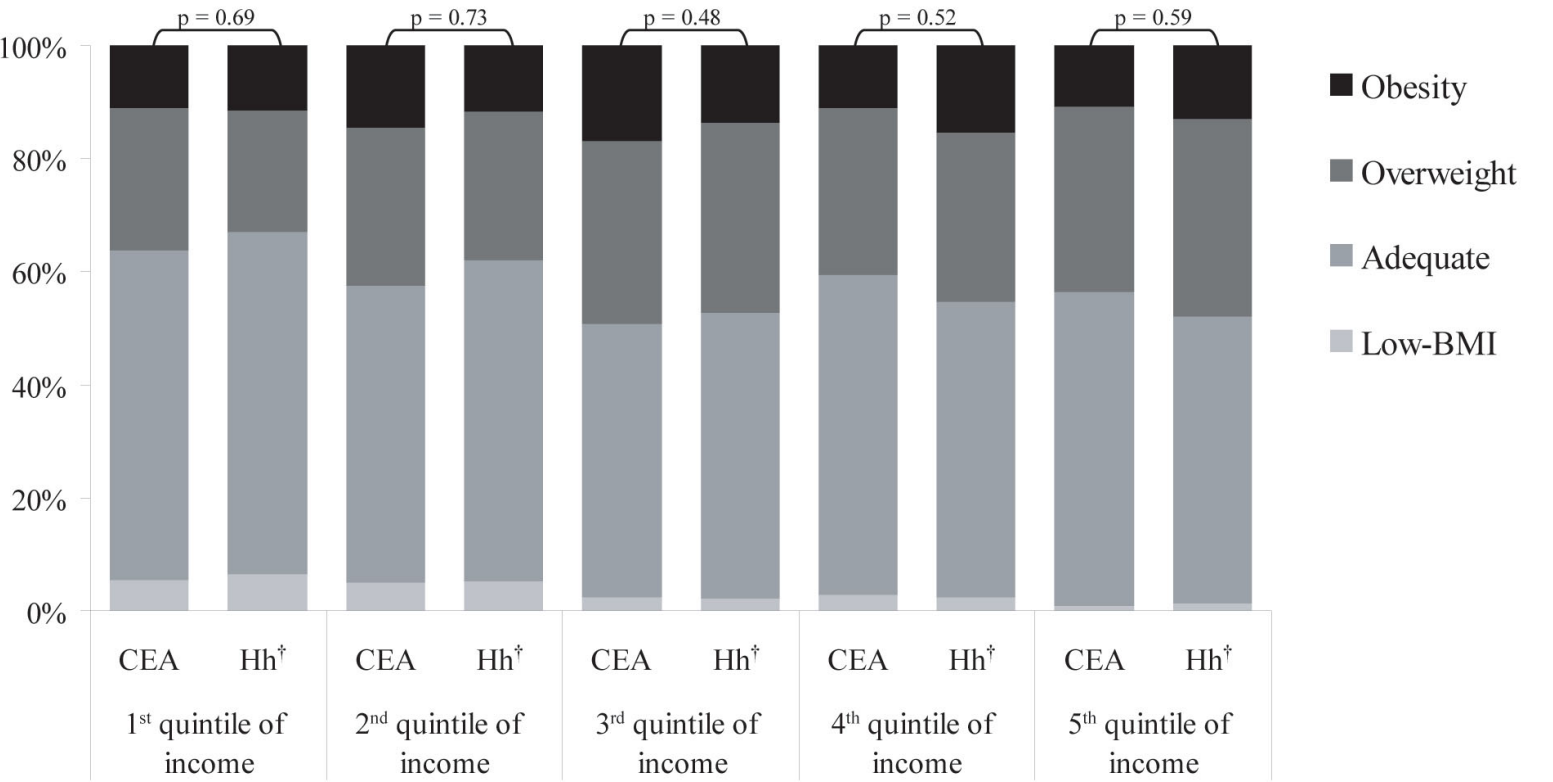
**Proportional distribution of the Niterói population (≥ 10 years of age), according to the nutritional status, by quintiles of household's monthly per capita income and quintiles of the head-of-household's mean nominal monthly income of the CEA**. ^†^Quintiles of household (Hh) income defined as the household's monthly per capita income. CEA = Census Enumeration Area (quintiles of CEA's income defined as the head-of-household's mean nominal monthly income of the CEA). p = p-value obtained from the Wald statistics for homogeneity between pairs of proportion vectors according to the nutritional status by income quintiles (α = 0.05).

Figure 1 presents the proportional distribution of nutritional status categories for each income quintile defined according to the household's monthly per capita income and the head-of-household's mean nominal monthly income of the CEA. The homogeneity between the pairs of proportions' vectors of each income quintile indicates that the distribution of the nutritional status categories in all income quintiles does not differ significantly according to the source of income information (household or CEA) used in the definition of quintiles (Figure 1). In other words, from a multivariate perspective, there is not enough statistical evidence to reject the hypothesis that the proportional distribution of nutritional status categories within each quintile would be different according to the source of income information used to categorize the quintiles.

The results of studies that investigate the use of area-level socioeconomic information as proxy of household or individual information are still controversial regarding the agreement of income measures as well as the results produced by each measure when related with an outcome. This is expected because the analysed outcomes, methods employed in the definition of socioeconomic strata, and the partitioning criteria used to define the territories vary between studies [12,16,39].

On one hand, the literature indicates that the information of both levels can be used without jeopardizing the analyses on health inequities because they produce similar results [15,16,18]. On the other hand, there is also evidence that the use of area-level information could result in substantial errors in the classification of socioeconomic conditions and, therefore, could not predict certain health outcomes as well as individual-level information [10-14,17,19].

In the present study, the structure of agreement/disagreement between quintiles of household monthly per capita income and quintiles of the head-of-household's mean nominal monthly income of the CEA, as well as the results produced by these measures when they were associated with the nutritional status of the population of Niterói, showed that the CEA's income information can be used when income information at the individual or household level are not available.

The hypothesis that the sex of the head of the household would not influence the structure of agreement/disagreement of income categories could not be rejected. Other factors, such as race [10,39], that could influence this structure were not analyzed in the present study. Another limitation of this analysis consists of the definition of partitioning of income strata in quintiles. This procedure was adopted based on an applied criterion, with an analytic purpose, since partitions with large intervals are generally used only with the purpose of planning sample and/or study design. However, it is important to register that partitions in larger intervals could have led to different conclusions because the distance between categories would be reduced, which could result in greater classification errors. On the other hand, smaller partitions -- in thirds, for example -- would increase the distance between categories, diminishing the chances of occurrence of misclassifications.

It is also important to pay attention to the time between when the information were assessed. This is particularly important in countries undergoing fast economic growth or greater socioeconomic mobility. The survey used in the present analysis was conducted only three years after the 2000 Brazilian census [9]. During this three-year period, the Brazilian economy was stable due to inexpressive and unsustained economic growth, and socioeconomic mobility was also compromised by declining gross domestic product, wage-share and continuous reduction of the formal employment sector.

Additionally, it is also important to note that the aggregated census information comes from individually collected information, which may raise the question whether the individual information collected in the census is reliable. The census information cannot be regarded as gold standard but it is expected to constitute more robust information than that collected in surveys because there are many more quality control mechanisms, proportionally fewer missing values, higher trust in the interviewer as an employee of a known institution (the Census Bureau), and no variance due to sample design.

Another issue that could be raised when dealing with aggregated data is that the income distribution within a given aggregated level (e.g., CEA, city) may vary according to the distance from a predetermined center. However, the adjusted models have not taken into account the modifiable areal unit problem and ecological fallacies due to aggregation [40-42], a limitation of the present study due to the absence of information on the distance between households and a predetermined center.

Furthermore, the inference and conclusions of this study may not apply to different variables of interest, countries, sizes and boundaries of enumeration areas, and possibly survey designs. Therefore, comparisons by other studies should be carefully made, taking this limitation into account.

## Conclusion

It is remarkable that until this paper, the few studies on this theme had been solely derived from high-income countries (United States of America [10-12], Canada [13-16], Italy [17], Spain [18] and Australia [19]). For this reason, it would be important to encourage other analyses using different levels of aggregation and territories in low- and middle-income countries such as Brazil, which would make intra- and international comparison possible, contributing to the collection of evidence about the use of socioeconomic information aggregated by CEA in the absence of individual information.

This is perhaps the first study conducted in a developing country that compares the use of area- versus household-level income measures in association with a health outcome (nutritional status). The study indicates that CEA's income information may be used as a proxy for household income in the absence of individual- or household-level information. The sex of the source of household income information did not influence the structure of agreement/disagreement of income categories. Additionally, the association between income quintiles and nutritional status is similar whether CEA- or household-level income measures were used.

## Competing interests

The authors declare that they have no competing interests.

## Authors' contributions

LAA and MTLV planned the research. MTLV designed the sample. FSG and MTLV calculated the natural and calibrated sampling weights and performed the statistical analysis. FSG wrote the first draft of the paper, which was revised and approved by the other authors.
